# Nonsurgical Management of Nifedipine Induced Gingival Overgrowth

**DOI:** 10.1155/2014/741402

**Published:** 2014-08-03

**Authors:** George Sam, Staly Chakkalakkal Sebastian

**Affiliations:** ^1^Department of Periodontics, Government Dental College, Kottayam, Kerala, India; ^2^Obstetrics and Gynaecology, Kerala Institute of Medical Sciences, Trivandrum, Kerala, India

## Abstract

Drug-induced gingival overgrowth is frequently associated with three particular drugs: phenytoin, cyclosporin, and nifedipine. As gingival enlargement develops, it affects the normal oral hygiene practice and may interfere with masticatory functions. The awareness in the medical community about this possible side effect of nifedipine is less when compared to the effects of phenytoin and cyclosporin. The frequency of gingival enlargement associated with chronic nifedipine therapy remains controversial. Within the group of patients that develop this unwanted effect, there appears to be variability in the extent and severity of the gingival changes. Although gingival inflammation is considered a primary requisite in their development, few cases with minimal or no plaque induced gingival inflammation have also been reported. A case report of gingival overgrowth induced by nifedipine in a patient with good oral hygiene and its nonsurgical management with drug substitution is discussed in this case report.

## 1. Introduction

Gingival enlargement is a well-known consequence of the administration of some anticonvulsants, immunosuppressants, and calcium channel blockers and may create speech, mastication, tooth eruption, and aesthetic problems.

Not all the patients using these agents are affected by gingival overgrowth, and the extent and severity are variable in such patients. Phenytoin-induced overgrowth may be present in 50 to 100% of patients treated with such drug, whereas cyclosporin and calcium channel blocker-induced overgrowths seem to be less common, with a prevalence of 30% and 20%, respectively [1–3]. Although there are previous reports of nifedipine induced gingival enlargement managed with nonsurgical therapy, there are no comprehensive description of cases managed effectively with drug substitution. This may partly be explained due to the enlargement in most cases having a predominant inflammatory component that often requires only an improvement in plaque control. In other cases, the present medical condition may prevent the offending drug from being discontinued. In the present case, the patient presented with minimal plaque and calculus suggesting a minor role of inflammation in the overall development of the enlargement. Since scaling and root planning did not show improvement in the condition, drug substitution was done with losartan potassium and the two months followup showed significant reduction in gingival enlargement.

## 2. Case Report

**Figure 1:**
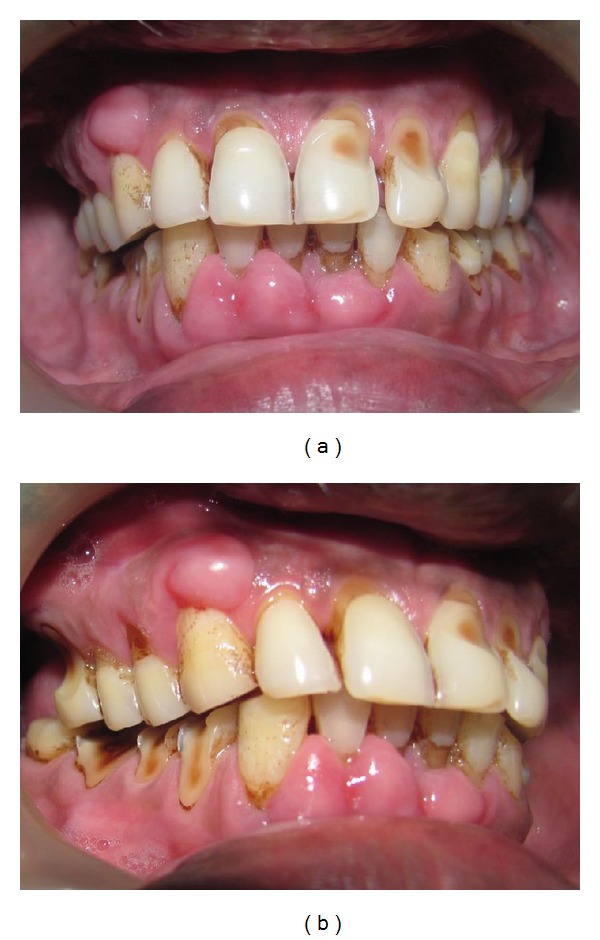
(a) Preoperative view. (b) Preoperative view.

**Figure 2:**
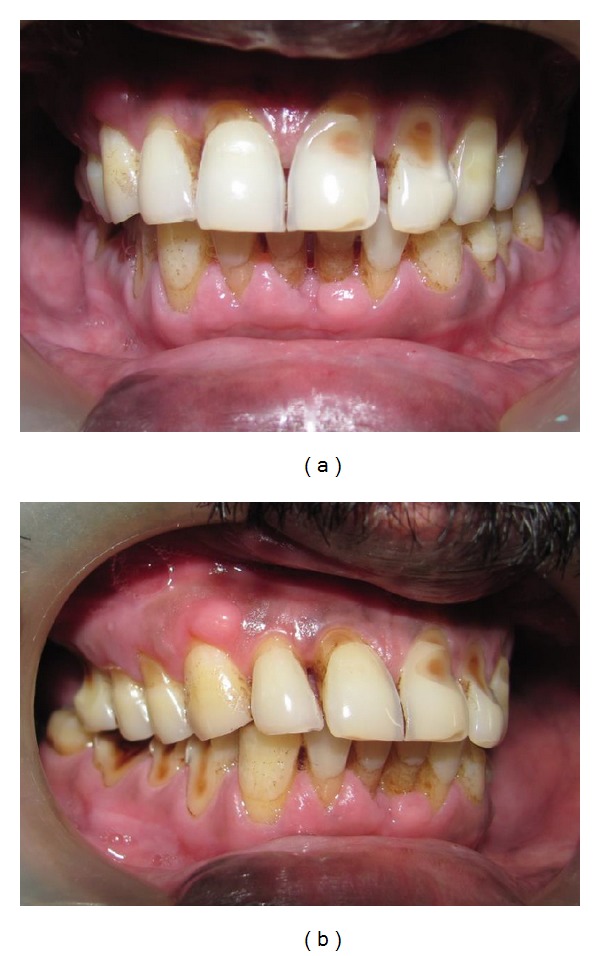
(a) Postoperative view (2 months after drug substitution). (b) Postoperative view (2 months after drug substitution).

A 53-year-old male patient reported to the Department of Periodontology, with a complaint of swollen gums. On examination, generalized gingival enlargement was noticed in the lower arch, whereas an isolated nodular growth was observed in the right side of upper arch. The enlarged gingiva was firm, pale pink, and resilient with a minutely lobulated surface and displayed no tendency to bleed (Figure 1). The teeth displayed generalized cervical abrasion, probably attributed to the vigorous tooth brushing habit of the patient. There were little amounts of calculus present, and no deep periodontal pockets were detected. The medical history of the patient revealed that the patient was hypertensive and that he was under medication for a period of 4 years for the same. He was consuming nifedipine 20 mg a day for the past 4 years. Based on the clinical presentation of the gingival enlargement and a history of nifedipine intake, the case was diagnosed as Nifedipine induced gingival overgrowth. Periodontal management consisted of performing thorough oral prophylaxis followed by careful instructions on oral hygiene procedures. The case was reviewed for any signs of improvement after a period of 2 weeks. Since there were no changes noticed, a referral was made to the patient's physician to consider drug substitution with respect to nifedipine. Nifedipine was substituted with losartan potassium 25 mg by the physician and the patient was reevaluated after 2 months. The bulk of the gingival enlargement had subsided in the lower arch and the isolated nodular growth in the upper arch had also reduced in size (Figure 2).

## 3. Discussion

The pathogenesis of drug-induced gingival overgrowths is still not completely understood. It has been demonstrated that gingival enlargement has a multifactorial nature and is affected by factors such as age, demographic variables, genetic predisposition, oral hygiene status, pharmacokinetic variables, and molecular and cellular changes in gingival tissues [4].

Despite their pharmacological diversity, the three major drugs causing gingival overgrowth, namely, anticonvulsants, calcium channel blockers, and immunosuppressants, have similar mechanism of action at the cellular level, where they inhibit intracellular calcium ion influx. The action of these drugs on calcium and sodium ion flux may prove to be the key in understanding why three dissimilar drugs have a common side effect upon a secondary target tissue, such as gingival connective tissue.

Calcium channel blockers are drugs developed for the treatment of cardiovascular conditions such as hypertension, angina pectoris, coronary artery spasms, and cardiac arrhythmias. Gingival enlargement associated with nifedipine was first reported in the early 1980s and was soon also described with diltiazem and verapamil and in cases with amlodipine and felodipine [4–6]. The possible hypothesis to explain this overgrowth is that the fibroblasts contain strongly sulfated mucopolysaccharides that are precursors of ground substance. After an interaction between nifedipine and gingival fibroblasts, overproduction of collagen and extracellular ground substance occurs and leads to an increase in the size of the gingiva. The drug interferes with the calcium metabolism of fibroblast cells and hence reduces the production of the degrading enzyme collagenase [7].

Some investigators believe that inflammation is a prerequisite for development of the enlargement, which therefore could be prevented by plaque removal and fastidious oral hygiene [8, 9]. The severity of gingival enlargement in patients taking medications correlates well with poor plaque control and is commensurate with the degree of plaque induced inflammation. This is supported by the fact that edentulous areas did not show signs of enlargement in most reported cases [10, 11]. A synergistic enhancement of collagenous protein synthesis by human gingival fibroblasts was found when these cells were simultaneously exposed to nifedipine and interleukin-1*β* (IL-1*β*), a proinflammatory cytokine that is elevated in inflamed gingival tissues [4]. But in our case there was little inflammation which is attributed to minimal amounts of plaque and calculus and good oral hygiene displayed by the patient. The enlargement had little relationship with inflammation, and other factors might have played a major role in their development.

It has also been proposed that susceptibility or resistance to pharmacologically induced gingival overgrowth may be governed by the existence of specific genetically predetermined subpopulations of fibroblasts in each individual which exhibit a fibrogenic response to these medications [4]. It has been suggested that there may be subpopulations of fibroblasts which are sensitive to nifedipine and cause an increase in the production of collagen [12].

Mast cells have been found to participate in many inflammatory oral diseases, particularly those associated with fibrosis. They possess very diverse roles ranging from proinflammatory to immunomodulatory. Upon their activation, they promote the local renin angiotensin system generation consequently able to stimulate endothelin and other profibrotic mediators [13].

The presence of the enlargement makes plaque control difficult, often resulting in a secondary inflammatory process that complicates the gingival overgrowth caused by the drug. The primary aim of nonsurgical approaches is to reduce the inflammatory component in the gingival tissues and thereby avoid the need for surgery. Patients at risk from or who have developed drug-induced gingival overgrowth will benefit from effective oral hygiene measures, professional tooth cleaning, scaling, and root surface instrumentation. For some patients these measures alone could reduce the gingival overgrowth to acceptable levels, and for others, it could make surgical correction easier [14–16]. However, in our case there was no resolution in the size of the enlargement following scaling and root planning because there was little inflammation to begin with. Nevertheless, the importance of strict plaque control in the management of drug-induced enlargement should not be underestimated.

The dose of the drug also has an impact on gingival oral growth. It is reported that nifedipine was found 15–316 times more in the gingival crevicular fluid compared to plasma [17]. The higher concentration of nifedipine in the gingival crevicular fluid could increase the severity of gingival enlargement [10]. Consideration should be given to the possibility of discontinuing the drug or of changing medication. These possibilities should be consulted with the patient's physician. Simple discontinuation of the offending agent is usually not a practical option but replacing it with another medication might be. Reduction in the size of the gingival overgrowth has been reported within a week of drug withdrawal and may lead to full resolution [18]. If any drug substitution is attempted, it is important to allow for 6–12 months to elapse between discontinuation of the offending drug and the possible resolution of gingival enlargement before a decision to implement surgical treatment is made [19].

The number of prescriptions for calcium channel blockers has been increasing in recent years. There is infinitesimal awareness about this effect of drugs on gingival tissues in medical community. There is a need for physicians and dentists to make a coordinated treatment plan for the patients indicated for these drug therapies. Our case showed that not every case of drug-induced gingival enlargement requires plaque induced gingival inflammation for their development. In such cases drug substitution should be considered a valid treatment option especially when the gingival enlargement is present in spite of good oral hygiene.
